# Mixture Toxicity in Human Health: Integrating One Health, Exposomics, and Modern Risk Assessment Strategies

**DOI:** 10.1021/acs.chemrestox.5c00375

**Published:** 2026-04-15

**Authors:** Jose L. Domingo, Martí Nadal

**Affiliations:** Laboratory of Toxicology and Environmental Health, School of Medicine, 73047 Universitat Rovira i Virgili, Sant Llorenç 21, Reus 43201 Catalonia, Spain

## Abstract

Human and environmental health are critically threatened by combined exposures to multiple chemical toxicants, including industrial chemicals, heavy metals, pesticides, endocrine-disrupting chemicals (EDCs), and per- and polyfluoroalkyl substances (PFAS). These substances interact biologically, producing additive, synergistic, or antagonistic effects that conventional single-substance risk assessments fail to predict. This leads to a systematic underestimation of health risks, particularly for vulnerable populations. Despite robust evidence on mixture toxicity, major regulatory frameworks such as the US Toxic Substances Control Act (TSCA) and the EU’s REACH program continue to assess chemicals in isolation. This review synthesizes current science on toxicant interactions and critiques global regulatory shortcomings, underscoring the real-world consequences through case studies on PFAS, heavy metals, and pesticide mixtures. It advocates for a paradigm shift, proposing reforms that integrate emerging tools like exposomics and computational toxicology with holistic frameworks such as One Health. We highlight pioneering regulatory efforts, including Canada’s mandate for cumulative risk assessments under CEPA and the EU’s development of mixture assessment factors (MAFs), as essential models for progress. Our recommendations include mandating science-based mixture assessments, harmonizing global standards, and implementing equity-driven policies to align regulations with the reality of multichemical exposures.

## Graphical abstract

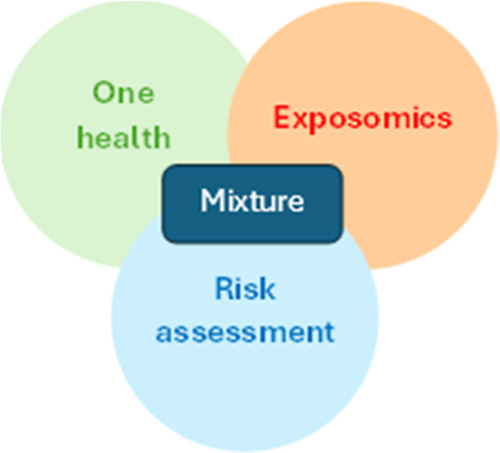

## 1 Introduction

The widespread presence of environmental toxicants, including industrial chemicals, heavy metals, pesticides, and persistent organic pollutants (POPs), poses important challenges to both human health and ecological integrity. Critically, organisms are rarely exposed to a single chemical in isolation. Rather, they continuously encounter complex mixtures of contaminants whose combined effects may differ substantially from those predicted by examining each substance individually. The WHO estimated that approximately 23% of global mortality is associated with environmental factors, with chemical exposures being a leading contributor.^1,2^ While this statistic reflects associations rather than definitive causal relationships and may not fully account for confounding factors such as socioeconomic status, it likely underestimates the true burden attributable to chemical exposures since regulatory risk assessments and epidemiological studies have traditionally evaluated compounds one at a time. This single-substance paradigm fails to capture the synergistic, additive, or antagonistic interactions that arise when multiple toxicants co-occur, a phenomenon collectively referred to as mixture toxicity, leaving a critical gap in the understanding of real-world chemical risk (see Tables 1 and 2).

**Table 1:** Overview of Key Regulatory Frameworks and Their Approach to Toxicant Mixtures

framework	jurisdiction/scope	mixture focus	key limitations	reform potential
Stockholm Convention on POPs	global	none; lists individual POPs for restriction/elimination	ignores co-occurrence and combined effects of POPs	broader consideration of chemical classes and cumulative exposures
Minamata convention on mercury	global	none; focuses solely on mercury	no consideration of mercury interactions with other neurotoxicants	integrate with broader neurotoxicant reduction strategies
SAICM/global chemicals framework	global	acknowledges mixtures as a concern	lacks binding mandates for mixture assessment	post-2020 framework to strengthen mixture-focused actions
US TSCA	USA	limited; single-substance risk evaluations	no routine mixture assessment; narrow “conditions of use”	use existing authorities for cumulative assessments
EU REACH	EU	limited; mixture assessment factors (MAFs) under development	MAFs not consistently applied; single-substance focus	implement MAFs and grouping strategies for mixtures
Canadian CEPA	Canada	emerging; 2023 reforms include cumulative effects	implementation details evolving	bill S-5 (2023) mandates cumulative effects assessment
food safety (e.g., pesticide MRLs)	national/regional	none; MRLs for individual pesticides	ignores combined effects of pesticide residues	cumulative risk assessment for pesticide groups
drinking water standards (MCLs)	national/regional	limited (e.g., total trihalomethanes)	fails to address contaminant cocktails	monitor emerging contaminants and mixture effects
air quality standards	national/regional	limited; standards for individual pollutants	ignores interactive effects of particulate matter (PM) and gaseous pollutants	research specific air pollution mixture effects

**Table 2:** Case Studies of Toxicant Mixtures and Regulatory Deficiencies

case study	toxicants and interactions	exposure routes and populations	health/environmental concerns	regulatory gaps
PFAS and EDCs	multiple PFAS (e.g., PFOA, PFOS); synergy with EDCs (e.g., BPA)	drinking water, food packaging; widespread exposure	endocrine disruption, developmental toxicity, carcinogenicity	TSCA/REACH: slow PFAS class regulation; single-substance focus
heavy metals	Pb, Cd, As, Hg; additive/synergistic neurotoxicity	water, soil, food; children vulnerable	neurodevelopmental deficits, kidney damage, cancers	clean water/SDWA: Individual limits miss mixture effects
pesticide mixtures	organophosphates, carbamates; synergistic neurotoxicity	diet, water, occupational; farmworkers	neurotoxicity, endocrine disruption, pollinator harm	FIFRA/EFSA: MRLs ignore dietary mixture effects

Human biomonitoring studies, such as the US National Health and Nutrition Examination Survey (NHANES) and the European Human Biomonitoring Initiative (HBM4EU), have demonstrated that individuals in contemporary societies harbor a complex “body burden” of hundreds of synthetic chemicals.^3,4^ These exposures are chronic and multipathway, occurring through inhalation, ingestion, dermal absorption, and occupational contact, and reflect the growing influence of the chemical exposome, the totality of environmental chemical exposures from conception onward.

Crucially, these exposures do not occur in isolation. A growing body of evidence reveals that toxicant mixtures can produce combined effects that differ significantly from those of individual substances considered alone.^5–7^ It is important to acknowledge that while synergistic interactions receive considerable attention in mixture toxicology research, most experimental evidence at environmentally relevant doses supports additive rather than synergistic effects, with true synergism documented in approximately 7% of binary mixtures studied.^7,8^ However, even additive effects represent a significant concern given that current regulatory frameworks may not adequately account for cumulative exposures. For example, combinations of per- and polyfluoroalkyl substances (PFAS) with bisphenol A (BPA) have been shown to enhance endocrine-disrupting activity beyond the effects of either compound alone.^9–11^ It should be noted that PFAS comprise hundreds or thousands of individual chemicals rather than a single compound, and real-world PFAS exposure typically involves multiple compounds simultaneously. In contrast, coexposures to lead (Pb), mercury (Hg), and cadmium (Cd) may amplify neurotoxic effects, particularly in children, whose developing nervous systems are highly vulnerable.^12^ Mixtures of organophosphate and carbamate pesticides can also exhibit additive or potentially synergistic neurotoxicity due to shared mechanisms of acetylcholinesterase inhibition. However, it should be noted that organophosphates typically form stable, often irreversible complexes with acetylcholinesterase, while carbamate inhibition is usually transient and reversible, affecting the duration and severity of observed effects.^13,14^

These mixture effects raise particular concern for vulnerable populations such as infants and children, pregnant women, the elderly, and low-income or marginalized communities, who may experience heightened biological sensitivity alongside disproportionate exposure burdens.^15,16^ Such populations frequently live in areas with high environmental contamination, have limited access to healthcare, and are often excluded from policymaking processes that affect environmental health protections.^17,18^

Despite mounting scientific evidence, the understanding of toxicant mixtures remains limited.^19–21^ Research in this area is constrained by the vast number of possible chemical combinations, the lack of real-world exposure data, and the high cost and complexity of experimental mixture studies.^22–24^ Although biomonitoring techniques can detect the presence of multiple chemicals in human tissues, relatively few studies have quantified their combined biological effects, especially at the low doses typical of chronic exposures.^25,26^ Additionally, epidemiological studies are often hindered by confounding variables, and global biomonitoring efforts remain uneven, with limited data available for many emerging contaminants such as novel flame retardants, plasticizers, nanomaterials, and transformation products of existing chemicals.^27–29^

A persistent gap remains between scientific insights into mixture toxicity and the regulatory systems addressing it.^30,31^ Most national and international frameworks, including the US Toxic Substances Control Act (TSCA), the EU REACH (Registration, Evaluation, Authorization and Restriction of Chemicals) regulation, and WHO guidance, still evaluate chemicals individually.^20,32–35^ Although agencies such as the US Environmental Protection Agency (EPA), WHO/IPCS, and ATSDR (Agency for Toxic Substances and Disease Registry) have developed mixture-assessment guidelines since the 1980s, their practical use remains limited. Under TSCA, risk evaluations rarely consider mixtures, except in specific cases such as intentional formulation or shared mechanisms of toxicity (e.g., organophosphate pesticides).^36,37^ This narrow focus overlooks the complex exposures occurring in real-world contexts. REACH has introduced the concept of Mixture Assessment Factors (MAFs), but these tools are still being refined, with implementation for high-priority substances expected in 2026.^38,39^ Similarly, international treaties such as the Stockholm and Minamata Conventions continue to target single substances, despite clear evidence of coexposure and possible interactions among contaminants in the environment and humans.^40,41^

The Food Quality Protection Act (FQPA) of 1996 represents a notable advance in US pesticide regulation, mandating that the US EPA ensures pesticides used on food are “safe”, with particular provisions for children. This includes an additional safety factor to account for uncertainties in data related to children’s possible susceptibility. Importantly, US EPA considers the combined effects of multiple pesticides that are judged to be toxicologically similar under FQPA, such as organophosphate and carbamate pesticides with common mechanisms of action. If multiple pesticides with shared toxicity pathways are present on food, their combined residue levels are considered when setting tolerances.^42,43^ Similarly, regulatory standards for food and water, such as acceptable daily intakes (ADI) and acute reference doses (ARfD) under the Federal Insecticide, Fungicide, and Rodenticide Act (FIFRA), or contaminant thresholds in the Safe Drinking Water Act, typically address one compound at a time, overlooking the reality of multiple residues and copollutants.^44,45^

This predominantly single-substance paradigm can contribute to potential underestimation of health risks posed by real-world exposures, particularly where synergistic interactions can increase toxicity at doses considered safe in isolation.^46^ Furthermore, the absence of harmonized global standards for mixture assessments impedes coordinated international responses and exacerbates health inequities.^47^

This review critically examines current regulatory approaches to addressing toxicant mixtures. It aims to (1) evaluate the limitations of prevailing single-substance approaches, (2) highlight the scientific basis for mixture-focused risk assessment, (3) illustrate potential health and environmental consequences of limited mixture consideration, and (4) propose evidence-based policy recommendations for reform. Furthermore, the present review identifies and examines pioneering regulatory developments, such as Canada’s operationalization of cumulative risk assessments under the Canadian Environmental Protection Act (CEPA) and the European Union’s planned implementation of MAFs under REACH. These initiatives provide critical, real-world models for how regulatory systems can begin to address the complexity of mixture exposures.

## 2 Search Strategy

A literature search was conducted to identify peer-reviewed studies, official reports, and policy documents published between January 1, 1980 and July 31, 2025. The starting date was expanded to 1980 to capture foundational work in mixture toxicology, including the seminal 1986 US EPA Guidelines for the Health Risk Assessment of Chemical Mixtures and the 2000 Supplementary Guidance for Conducting Health Risk Assessment of Chemical Mixtures, which provide methodological foundations for evaluating human health risks from environmental chemical mixtures. Searches were performed across major scientific databases, including PubMed, Scopus, Google Scholar and Web of Science, and using Boolean combinations of keywords. Specific search terms included: “toxicant mixtures”, “chemical mixtures”, “combined effects”, “cumulative exposure”, “regulatory gaps”, “chemical policy”, “PFAS”, “endocrine disruptors”, “heavy metals”, “pesticides”, and “persistent organic pollutants”.

Example search strings included: (“toxicant mixtures” OR “chemical mixtures”) AND (“regulation” OR “policy”) AND (“health effects”); (“PFAS” OR “perfluoro”) AND (“mixture” OR “combined exposure”); (“heavy metal” OR “lead” OR “mercury” OR “cadmium”) AND (“mixture” OR “coexposure”) AND (“neurotox” OR “developmental”). These were used to capture literature on both health impacts and regulatory responses. To expand coverage, reference lists of key articles and reviews were screened manually, while major agency websites (e.g., US EPA, ECHA, EFSA, WHO, UNEP) were consulted for relevant documents. Inclusion criteria focused on studies addressing mixture toxicity mechanisms, health or environmental impacts, regulatory analyses, and proposed policy reforms. Only English-language sources were included. Exclusion criteria ruled out non-peer-reviewed sources (except official reports) and articles focused solely on single-substance toxicity. Study quality was evaluated using a framework considering methodological rigor (study design, sample size, statistical methods), peer-review status, and relevance to regulatory applications. Higher weight was given to systematic reviews, meta-analyses, and studies with robust exposure assessment and control for confounding variables. Relevant findings were synthesized narratively to evaluate regulatory shortcomings, highlight scientific challenges, and support evidence-based policy recommendations. The key themes presented in the Results section were identified through an iterative thematic synthesis approach. After the initial literature search, both authors independently screened titles and abstracts for relevance. Full-text articles that met the inclusion criteria were then examined in detail, allowing the identification and coding of recurring topics concerning regulatory gaps, mechanisms of mixture toxicity, challenges in exposure assessment, and recent policy developments. The thematic categories were subsequently refined through discussion and cross-referencing with relevant regulatory and scientific documents. Themes were retained when they appeared consistently across multiple independent sources, including systematic reviews, primary studies, and agency reports. This synthesis process informed both the structure of the Results section and the formulation of the policy recommendations presented in this work.

## 3 Results

The literature review highlights major themes in the science and regulation of chemical mixtures. Evidence shows that real-world exposures involve complex combinations of chemicals, whose collective effects are often underestimated by single-substance regulatory models. This synthesis first critiques existing frameworks and their limitations, then outlines current knowledge of toxicant interaction mechanisms (molecular and toxicokinetic bases of additive, synergistic, and antagonistic effects) and the key challenges of mixture risk assessment. It closes with case studies on PFAS, heavy metals, and pesticide mixtures and discusses emerging tools and policy options for future reform.

### 3.1 Current Regulatory Frameworks: A Predominant Single-Substance Focus

Despite decades of evolving chemical safety legislation and a growing scientific understanding of the complexities of chemical exposures, most national and international regulatory systems remain firmly anchored to a paradigm that assesses and manages chemicals as isolated, individual entities.^48,49^ This single-substance focus inherently fails to account for the common reality of concurrent exposures to multiple chemicals and their potential interactions. An examination of key global and national frameworks reveals ongoing challenges in addressing mixture toxicity.^50^

Internationally, prominent treaties and agreements designed to manage hazardous chemicals, such as the Stockholm Convention on POPs (2001), the Rotterdam Convention on Prior Informed Consent (1998), the Basel Convention on the Control of Transboundary Movements of Hazardous Wastes and Their Disposal (1989), and the Minamata Convention on Mercury (2013), primarily target specific, high-concern chemicals or defined waste streams. While crucial for controlling some of the most dangerous substances, these conventions universally adopt a single-substance approach.

The Comprehensive Environmental Response, Compensation, and Liability Act (CERCLA), which addresses the cleanup of hazardous waste sites in the United States, includes approaches for evaluating chemical mixtures at Superfund sites, representing one of the earlier regulatory frameworks to acknowledge mixture exposures in contaminated environments. The ATSDR has developed frameworks for mixture assessment under CERCLA, including a binary weight-of-evidence (BINWOE) classification scheme and approaches for pooling target organ outcomes. Nevertheless, these methods have limitations, such as focusing on pairwise interactions within complex mixtures and potentially overlooking toxicokinetic differences that can affect sites of action. The Stockholm Convention, for example, lists POPs individually for restriction or elimination, despite clear evidence of their co-occurrence in environmental matrices and biological samples, and the potential for combined effects. The Rotterdam and Basel Conventions focus on the international trade of hazardous chemicals and the management of hazardous waste, respectively, but lack substantive provisions for assessing or mitigating risks arising from chemical mixtures within these contexts.^51^ Similarly, the Minamata Convention is dedicated to regulating Hg, a potent neurotoxicant, but does so in isolation, without considering its interactions with other co-occurring pollutants like lead or cadmium, which can exacerbate neurodevelopmental impacts. Even broader initiatives like the Strategic Approach to International Chemicals Management (SAICM) and its post-2020 framework (now the Global Framework on Chemicals—Toward a Planet Free of Harm from Chemicals and Waste) acknowledge the issue of mixture exposures but historically have lacked binding enforcement mechanisms to mandate their assessment or management.^52^

National regulatory frameworks in many industrialized nations mirror this international trend. In the United States, the TSCA, even after significant amendments in 2016 (the Frank R. Lautenberg Chemical Safety for the 21st Century Act), continues to direct the EPA to evaluate chemicals primarily based on their individual properties and “conditions of use”. While the amended TSCA mandates risk evaluations for existing chemicals and reviews for new chemicals, it does not explicitly require the assessment of risks from combined exposures to multiple chemicals, except in very limited circumstances.^37^ The European Union’s REACH regulation is often cited as one of the most comprehensive chemical management systems globally. However, REACH also predominantly focuses on single-substance registration and risk assessment dossiers submitted by manufacturers. While there has been ongoing discussion and development of methodologies like the use of MAFs to account for unintentional coexposures, these are not yet consistently or mandatorily applied across all relevant scenarios and remain a subject of ongoing scientific and policy development.^39^ Other national legislations, such as those in Japan and China, also tend to emphasize single-substance evaluations for chemical registration and control.

### 3.2 Notable Progress in Mixture Regulation

Despite the predominance of single-substance approaches, several promising regulatory developments merit recognition. Canada has emerged as a potential model for progress in addressing chemical mixtures. Since 2023, under the CEPA, Canada has implemented pilot programs to operationalize cumulative risk assessments, with specific focus on PFAS and pesticide mixtures.^53^ These initiatives represent some of the first mandatory requirements for mixture assessment within a major regulatory framework.

Similarly, the European Union’s Chemicals Strategy for sustainability (2020–2024) has advanced mixture considerations by mandating the inclusion of MAFs in REACH dossiers for high-priority substances, with enforcement scheduled to begin in 2026. This represents an important step toward acknowledging the “mixture reality” within the EU’s chemical management system. Recent methodological advances in dose addition understanding, including concepts such as convergence of toxicity pathways, have been documented in US EPA’s frameworks for mixture assessment, providing updated guidance that builds upon the foundational 1986 and 2000 US EPA mixture guidelines.

While in the USA, broad regulatory reform remains limited, targeted actions addressing specific mixture concerns have emerged. Notably, the US EPA finalized binding PFAS drinking water standards in 2024 (4–10 ppt for PFOA/PFOS), marking the first federal regulation explicitly acknowledging cumulative risks for PFAS mixtures. Notwithstanding, it should be noted that these standards have faced implementation challenges and partial modifications under current administration policies. These developments, while insufficient to address the full scope of mixture toxicity challenges, demonstrate feasible pathways for incorporating mixture considerations into existing regulatory frameworks.

This single-substance focus is also pervasive in sector-specific regulations. For example, food safety standards typically establish maximum residue limits (MRLs) for individual pesticides in various commodities. However, it is common for food items to contain residues of multiple pesticides, yet the regulatory limits are generally not adjusted to account for potential additive or synergistic effects of these co-occurring residues.^44^ In Europe, cumulative risk assessment of pesticide residues has been conducted using ADI and RfD values for groups of pesticides with common mechanisms, demonstrating the feasibility of mixture-based approaches, although implementation remains limited in scope. On the other hand, drinking water quality standards usually set maximum contaminant levels (MCLs) for individual chemical contaminants, with only a few exceptions for groups of related substances like total trihalomethanes (disinfection byproducts).^45,54^ Many epidemiological studies of dioxin-like compounds (DLC) use the toxic equivalency factor (TEF) approach along with measures of exposure to DLC mixtures, demonstrating the feasibility of mixture-based assessment approaches. Similarly, several drinking water disinfection byproduct (DBP) epidemiology studies evaluate developmental health effects from subsets of DBP mixtures, such as haloacetic acids or trihalomethanes, using relative potency factors for mixture analysis.^55^ Air quality standards also target individual criteria pollutants (e.g., ozone, sulfur dioxide, nitrogen oxides, and particulate matter), even though atmospheric pollution, particularly PM, consists of a complex mixture of numerous chemical species.^56^

Occupational exposure limits (OELs) in workplace settings are also typically set for individual chemicals, and while some guidance exists for assessing simple additive effects for chemicals with similar modes of action, more complex interactions like synergism are rarely accounted for in regulatory compliance.^57^ Consumer product regulations tend also to assess the safety of individual chemical ingredients rather than the potential effects of exposures to mixtures of chemicals leaching from products or arising from the use of multiple products concurrently.^58^

This siloed, chemical-by-chemical approach across various regulatory domains fails to capture the reality of multimedia and multipathway exposures that characterize modern life, potentially leading to an underestimation of overall chemical risk. This persistent focus on single toxicants creates challenges in regulatory oversight, that may leave the public and the environment inadequately protected from the combined effects of the chemicals to which they are exposed daily.

### 3.3 Scientific Understanding of Toxicant Interactions: Mechanisms, Challenges, and Data Gaps

Building upon the outlined regulatory landscape, it is essential to delve into the scientific basis for mixture toxicity. The interactions between different toxicants are complex and can significantly alter their individual effects, a reality that current single-substance regulatory models largely fail to capture. Understanding these mechanisms, the challenges in assessing them, and the existing data gaps is fundamental to appreciating the urgent need for regulatory reform.

### 3.4 Mechanisms of Interaction

Toxicant mixtures interact through additive, synergistic, or antagonistic mechanisms.^14,59–61^ Additive effects occur when chemicals with similar modes of action, such as neurotoxic metals (Pb, Cd, Hg), elicit cumulative toxicity via shared molecular targets. Synergistic interactions, in which combined effects exceed individual toxicities, are particularly concerning (for example, PFAS and BPA may jointly amplify endocrine disruption at low doses). Such synergism can arise when one compound impairs another’s detoxification (toxicokinetic synergy, as seen with organophosphates inhibiting carbamate hydrolysis), or when distinct pathways converge on common signaling nodes (toxicodynamic synergy). Antagonism, although less examined, occurs when one chemical reduces another’s toxicity, as with antioxidants mitigating metal-induced oxidative stress or competitive receptor binding. Interaction outcomes depend on dose, timing, exposure route, and susceptibility, complicating linear, single-chemical risk models.^62,63^ Independent action, where compounds act on separate targets with minimal interaction, often serves as a baseline assumption in mixture assessments.

### 3.5 The Evidence Base for Synergistic Effects

It is essential to acknowledge the ongoing scientific debate regarding the prevalence of synergistic interactions at environmentally relevant concentrations. While synergistic effects can occur under controlled experimental conditions, they cannot necessarily be representative of human-relevant exposure scenarios without supporting dose and timing data. A meta-analysis by Cedergreen^8^ examined 194 binary mixtures and found true synergism in approximately 7% of cases, with most mixtures exhibiting additive effects. This finding underscores the importance of not overemphasizing synergistic interactions while recognizing that even the documented cases of synergism represent significant concerns given the thousands of chemicals in commerce and the potential for serious health impacts when synergism does occur. There is a strong bias toward investigating additive and synergistic effects in the literature.^64^ Most investigations report synergism at concentrations exceeding typical exposure concentrations, while the relevance of exposure levels for the occurrence of synergism is an important issue. More recent studies have identified specific conditions that may promote synergistic interactions at lower, environmentally relevant concentrations. For example, Kortenkamp^65^ demonstrated synergistic effects between certain PFAS compounds and other endocrine-disrupting chemicals (EDCs) at concentrations within human exposure ranges, particularly when targeting specific endocrine pathways. Similarly, research on pesticide mixtures by Cedergreen^8^ reported synergistic interactions between organophosphates and certain fungicides at concentrations found in agricultural workers. However, few studies assess mixture effects at environmentally relevant levels, and existing evidence remains inconsistent. Although mechanisms of synergism are still being clarified, current knowledge indicates that such interactions, particularly among endocrine disruptors, developmental toxicants, and chemicals sharing target organs, deserve careful regulatory attention.

### 3.6 Challenges in Risk Assessment of Mixtures

Mixture risk assessment faces three core challenges: the vast number of possible chemical combinations, reliance on single-substance toxicological data poorly suited for predicting interactions, and nonlinear/nonmonotonic dose-responses (NMDRs), frequently observed with EDCs that complicate extrapolation from high-dose studies. New approach methodologies (NAMs) offer complementary strategies to address these limitations, encompassing in silico and in vitro methods, omics-based techniques, and alternative in vivo approaches.^66^ Tools such as quantitative structure–activity relationship (QSAR), physiologically based pharmacokinetic (PBPK) models, and high-throughput screening platforms show considerable potential for predicting mixture effects and reducing animal testing.^2,22–24,67,68^ Nevertheless, broader NAM adoption remains constrained by insufficient validation data, the inherent complexity of biological interactions, as well as the absence of comprehensive regulatory guidance for their implementation in mixture risk assessments.

### 3.7 Limitations in Exposure Science

Accurately characterizing human and environmental exposure to chemical mixtures, the exposome, is another major scientific hurdle. The exposome is dynamic and varies significantly based on geographical location, lifestyle, diet, occupation, age, and socioeconomic status.^69^ While biomonitoring studies (e.g., measuring chemical concentrations in blood or urine) can detect the presence of multiple toxicants in human bodies, providing direct evidence of coexposure, comprehensive biomonitoring programs that cover a wide range of chemicals and diverse populations are scarce, particularly in low- and middle-income countries.^26^ Furthermore, detecting chemicals does not automatically equate to understanding their combined health impact. Epidemiological studies attempting to link mixture exposures to health outcomes face significant methodological challenges, including accurately assessing past exposures, controlling for numerous confounding variables (e.g., genetics, lifestyle, socioeconomic factors), and disentangling the effects of individual components within a complex mixture.^27^

Global biomonitoring efforts are also uneven, with significant data gaps for many emerging contaminants of concern, such as novel flame retardants, plasticizers, nanomaterials, and transformation products of existing chemicals, further hindering comprehensive mixture risk assessments.^29^ Advances in high-resolution mass spectrometry (HRMS) and exposomic databases (e.g., the EU’s HBM4EU initiative) now enable untargeted screening of thousands of chemicals in biological samples, yet regulatory risk assessments lag in adopting these tools. Without robust exposure data, even the most sophisticated toxicological models for mixture effects cannot be effectively applied to real-world scenarios.

These scientific complexities and data limitations underscore the inadequacy of the current single-substance regulatory approach and highlight the urgent need for new methodologies and a paradigm shift in how we assess and manage the risks from chemical mixtures. A conceptual model illustrating the fundamental failure of current single-substance regulatory paradigms to adequately address the complexities of real-world toxicant mixture exposures is shown in Figure 1.

**Figure 1:**
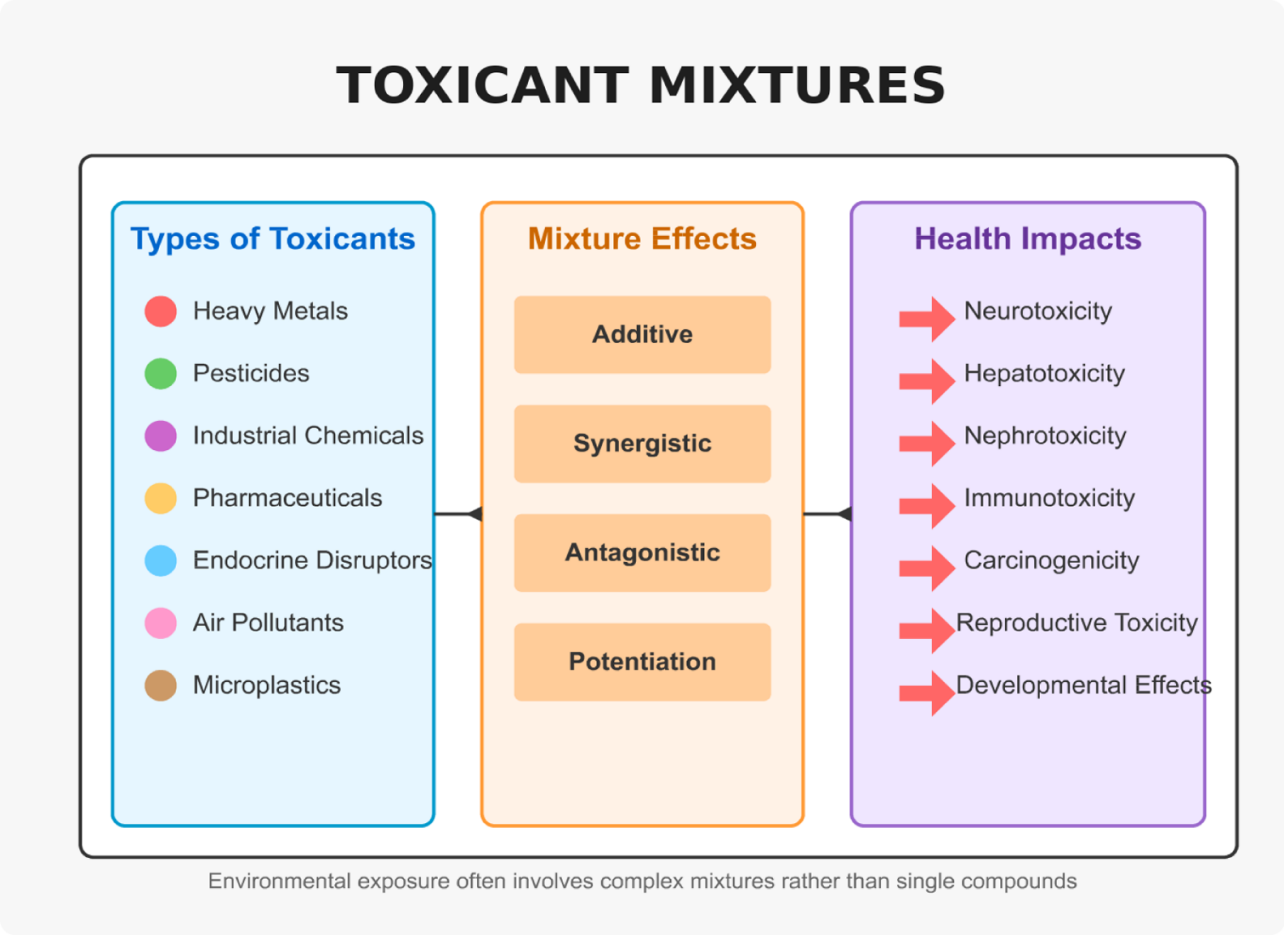
Conceptual model illustrating the fundamental failure of current single-substance regulatory paradigms to adequately address the complexities of real-world exposures to toxicant mixtures.

### 3.8 Regulatory Gaps and Weaknesses: Real-World Exposures

The preceding discussions on current regulatory frameworks and the scientific understanding of toxicant interactions clearly illuminate a fundamental challenge in how chemical risks are managed globally: the overwhelming reliance on a single-substance paradigm.^70^ This approach may systematically underestimate, and often entirely ignore, the health risks associated with cumulative and interactive exposures to multiple chemicals.^71^ This section will delve deeper into the specific regulatory gaps and weaknesses that arise from this predominant model.

### 3.9 The Pervasive Single-Substance Focus and Its Consequences

A significant regulatory gap is the near-universal focus on assessing chemicals one by one. Major regulatory frameworks like TSCA in the US, and REACH in the EU, along with international conventions, primarily evaluate the safety of individual substances against specific toxicity end points. This approach inherently fails to consider that in the real world, humans and ecosystems are never exposed to just one chemical at a time. Instead, they are exposed to complex mixtures from diverse sources including air, water, food, consumer products, and occupational environments. The assumption that regulating individual chemicals to their respective “safe” levels will automatically protect against the effects of mixtures cannot be scientifically supported, especially when synergistic interactions occur.^7^ This single-substance paradigm, therefore, may create a false sense of security and potentially lead to an underestimation of true public health and environmental risks.

### 3.10 Inconsistent Standards and Lack of Harmonization

Compounding the problem of the single-substance focus is the significant inconsistency in chemical standards and risk assessment methodologies across different jurisdictions and even between different regulatory programs within the same country. For example, acceptable exposure limits or regulatory thresholds for the same chemical (e.g., specific PFAS compounds, pesticides, or heavy metals) can vary substantially between the US, the EU, Canada, and other nations.^72–74^ These discrepancies make it exceedingly difficult to develop a coherent global or even regional strategy for managing mixture risks. If individual chemical standards are not harmonized, the foundation for assessing mixtures becomes even more fragmented and unreliable. This lack of harmonization complicates international trade, hinders collaborative research, and ultimately results in unequal levels of public health protection across different populations.

### 3.11 Insufficient Application of Precautionary Measures, Especially for Vulnerable Populations

Some regulations may not adequately apply the precautionary principle, which advocates for proactive measures to prevent harm even in the face of scientific uncertainty. This is particularly relevant for chemical mixtures, where the full extent of interactive effects is often unknown. Furthermore, regulatory standards are typically based on studies in healthy adult animals or average human populations, with safety factors applied to extrapolate to sensitive subgroups. However, these safety factors may not be sufficient to protect vulnerable populations, including fetuses, infants, children, pregnant women, the elderly, and individuals with pre-existing health conditions or genetic susceptibilities, from the effects of chemical mixtures.^16^ Children, for instance, have different exposure patterns, metabolic capacities, and developmental sensitivities compared to adults, making them uniquely vulnerable.^48^ Regulations rarely incorporate sufficiently protective, mixture-specific standards or lower exposure thresholds tailored to these vulnerable groups, potentially contributing to environmental injustice and health disparities.

### 3.12 Industry Influence and Significant Data Gaps

Regulatory processes can be influenced by industry interest, which can delay or weaken protective measures, including those related to mixture assessments. Manufacturers are typically required to submit toxicological data for individual substances they produce or import, but there is rarely a requirement to provide data on the potential interactions of their chemicals with other substances commonly found in the environment or in consumer products. This creates substantial data gaps regarding mixture toxicity.

In some regulatory contexts, the burden of proof can fall on regulatory agencies to demonstrate that a mixture is harmful, rather than on manufacturers to demonstrate that their chemicals are safe in the context of real-world mixed exposures. This reactive, rather than proactive, approach, coupled with the resource-intensive nature of mixture research, means that regulatory action on mixtures is often slow and insufficient. Data gaps are particularly acute for many PFAS mixtures, complex pesticide formulations, and the combined effects of EDCs.^73^ To address potential bias, enhanced mandates for independent, publicly funded mixture studies and transparent conflict of interest disclosures in regulatory submissions would help ensure more balanced scientific assessments.

### 3.13 Ethical and Legal Considerations

From an ethical standpoint, limited attention to mixture toxicity means that current regulatory systems may not be optimally fulfilling their primary mandate to protect public health and the environment.^23^ A more balanced approach might consider placing the burden of demonstrating safety in the context of combined exposures more heavily with the industries that produce and profit from these chemicals, aligning with principles of accountability and enhanced application of the precautionary principle.^34^

Current legal frameworks often lack clear mandates or effective enforcement mechanisms to compel comprehensive mixture assessments. While some laws, like FIFRA in the US, have provisions for cumulative risk assessment for pesticides with common mechanisms of toxicity, their application has been narrow and has not extended to the broader universe of chemical mixtures with dissimilar mechanisms that may still interact.^75^ These challenges perpetuate a system that cannot be optimally equipped to deal with the chemical realities of the 21st century.

### 3.14 Case Studies: Illustrating Challenges in Real-World Scenarios

To concretely illustrate potential consequences of the regulatory challenges discussed, this section presents case studies focusing on three classes of toxicants, PFAS, heavy metals, and pesticides, where mixture exposures are common and the limitations of single-substance approaches are particularly evident. These examples highlight potential gaps in how current regulatory systems address public health and environmental protection from the combined effects of chemicals.

#### 3.14.1 Case Study 1: Per- and Polyfluoroalkyl Substances (PFAS) and Co-Occurring Endocrine Disruptors

PFAS are a large family of synthetic chemicals characterized by their extreme persistence in the environment and the human body.^76^ They are found in industrial and consumer products, leading to widespread human exposure, primarily through contaminated drinking water and food. Biomonitoring studies detect multiple PFAS congeners in human blood and milk, indicating ubiquitous coexposure.^77,78^ At the molecular level, PFAS exert their endocrine-disrupting effects primarily through activation of peroxisome proliferator-activated receptors (PPARs), competition with thyroid hormone and fatty acid-binding proteins, and modulation of nuclear receptor signaling. When co-occurring with other EDCs such as bisphenol A (BPA), which acts via estrogen receptor (ERα) pathways, these distinct but interconnected molecular targets can produce convergent downstream effects on hormonal homeostasis. Research has shown that PFAS may act synergistically with other EDCs like BPA, for example, potentially enhancing thyroid disruption or other endocrine-mediated effects at concentrations where individual chemicals might show minimal impact.^79^ However, the concentrations and test systems used in these studies should be carefully evaluated for their environmental relevance and applicability to human health at realistic exposure levels.

Regulatory responses have been slow and fragmented. In the US, TSCA has faced challenges in effectively regulating existing PFAS or preventing new, problematic PFAS from entering the market. While the US EPA has recently taken steps to establish national drinking water standards for a few PFAS, these efforts have been long delayed, and the single-substance approach still largely does not address the risks from the thousands of other PFAS or their combined effects with other EDCs. Communities near industrial facilities or military bases using PFAS-containing firefighting foams often face disproportionately high exposures and potential health risks.^80^ Recent litigation, such as the 2024 settlement with 3M over PFAS contamination, underscores growing legal pressure to address mixture risks, though regulatory frameworks remain reactive rather than preventive. Epidemiological studies show associations between PFAS exposure and various health outcomes including cancer, liver damage, and immune system dysfunction, although these associations may not definitively establish causation due to potential confounding factors. This case demonstrates potential challenges when chemical classes are not addressed as mixtures, potentially resulting in widespread contamination, costly remediation, and possible public health impacts.

#### 3.14.2 Case Study 2: Heavy Metals—The Flint Water Crisis and Beyond

The coexposure to multiple heavy metals, such as Pb, Cd, arsenic (As), and Hg, is a significant public health concern globally. These metals can contaminate drinking water, soil, and food sources. Children are particularly vulnerable to the neurotoxic effects of heavy metals, partly because developing neural tissue expresses high levels of metal transporters and is more permeable to ionic species crossing the blood–brain barrier. At the molecular level, several mechanisms underlie the additive and synergistic neurotoxicity observed in heavy metal mixtures. Lead and Hg both disrupt glutamate signaling and inhibit *N*-methyl- *d* -aspartate (NMDA) receptor function, impairing synaptic plasticity through convergent pathways. In turn, Cd competes with zinc and calcium for binding sites on metallothioneins and calmodulin-dependent enzymes, amplifying oxidative stress when combined with Pb or Hg by depleting antioxidant defenses. Arsenic further contributes through inhibition of sulfhydryl-dependent enzymes and interference with DNA methylation, which may synergize with Pb-induced epigenetic disruption during critical developmental windows. It has been shown that coexposure to various toxic elements may exacerbate toxic effects, with their combined impact potentially being greater than the sum of their individual toxicities.^81–84^ The Flint, Michigan water crisis serves as an example of challenges in addressing multiple chemical exposures.^85,86^ While the primary focus was on lead contamination resulting from corrosive water leaching lead from aging pipes, the situation also involved other water quality issues and potential coexposures, including harmful disinfection byproducts and bacterial contaminants (*Legionella*). In this case, the specific potentially interactive effects of the chemicals contaminating the water system warrant detailed analysis to understand the full scope of mixture impacts.

The Clean Water Act and Safe Drinking Water Act in the US set individual standards for contaminants but generally do not account for the potential for interactive effects when multiple metals or other toxicants are present simultaneously.^87^ This case highlighted challenges in regulatory oversight, gaps in testing for mixtures, and a delayed response that disproportionately affected a vulnerable, predominantly low-income and minority community. Studies suggest associations between persistent cognitive deficits in Flint children with combined lead–cadmium exposure, although these associations require careful interpretation considering potential confounding factors, reinforcing the need for mixture-specific response policies.^88,89^ These findings highlight the critical need for environmental justice considerations in both regulatory frameworks and remediation efforts.

#### 3.14.3 Case Study 3: Pesticide Mixtures in Food and the Environment

Modern agricultural practices result in complex pesticide mixtures as residues in food and water, exposing consumers through diet and agricultural workers through occupational contact.^14,90^ Organophosphates and carbamates share a common neurotoxic mechanism (acetylcholinesterase (AChE) inhibition) but differ critically in reversibility: organophosphates form stable, often irreversible enzyme complexes, whereas carbamates produce spontaneously reactivating carbamylated complexes. Therefore, coexposure can generate toxicokinetic and toxicodynamic synergism, with organophosphate-induced inhibition prolonging carbamate effects. In addition, azole fungicides inhibiting cytochrome P450 (CYP) enzymes may impair organophosphate detoxification, amplifying systemic toxicity below individually harmful thresholds. Beyond neurotoxicity, pesticide mixtures may interact across endocrine, developmental, and immune systems through distinct mechanisms, producing combined adverse effects that current regulatory frameworks may not fully capture.

Regulatory frameworks like the US FIFRA and European food safety regulations (EFSA) primarily set ADI and ARfD for individual pesticides. While there have been efforts to implement cumulative risk assessments for groups of pesticides with common mechanisms of toxicity (e.g., organophosphates), these assessments are often limited in scope, slow to be completed, and do not typically address mixtures of pesticides with different mechanisms of action or the combined effects of pesticides with other environmental toxicants.^14,44,91^ Furthermore, these regulations often do not account for the potential cumulative effects where low-level exposures to multiple residues, each within legal limits, may collectively pose elevated health risks. Recent studies have demonstrated that bees exposed to field-realistic mixtures of neonicotinoids and fungicides experience significantly greater mortality and behavioral disruptions than predicted by individual assessments, although the relevance to human health requires further investigations.^92^

These case studies underscore a consistent pattern: regulatory systems designed around single-chemical assessments may not be optimally equipped to address the complex reality of mixed exposures, potentially leading to an underestimation of risks and challenges in protection of public and environmental health.

## 4 Discussion

The analysis presented in this review identifies a significant misalignment between the scientific understanding of toxicant mixture exposures and the prevailing regulatory paradigms worldwide. The evidence suggests: humans and ecosystems are ubiquitously exposed to complex mixtures of chemicals, not isolated substances. These mixtures can interact in additive, synergistic, or antagonistic ways, often leading to health and environmental outcomes that are not predictable from single-substance assessments alone.^46,62^ Despite mounting scientific evidence dating back decades, the dominant regulatory approach remains anchored in a predominantly chemical-by-chemical assessment strategy, an approach that may not be optimally suited to our chemically complex world.

This gap, as illustrated by the examination of international treaties, national regulations (including TSCA and REACH), and sector-specific policies, can result in potential underestimation of risks and, consequently, challenges in providing optimal protection for public health and the environment. The case studies on PFAS, heavy metals, and pesticides demonstrate potential implications of these regulatory challenges, highlighting how vulnerable populations often bear a disproportionate burden of these potentially unaddressed risks.

The regulatory gaps identified in this review represent significant challenges rather than simple oversight. These include the pervasive single-substance focus, inconsistent standards across jurisdictions, insufficient application of precautionary measures, potential industry influence, and significant data deficiencies, all of which create a regulatory landscape that cannot optimally prioritize comprehensive public health protection. While it is important to note that many individuals are living healthier and longer lives compared to previous generations, these gaps, nonetheless, translate into important public health considerations, including potential increased risks of endocrine disruption, neurodevelopmental disorders, various cancers, and other chronic diseases. The challenge in mandating comprehensive mixture assessments before chemicals are allowed on the market or before exposure limits are set means that society is often reacting to problems only after harm has occurred, rather than proactively preventing it. This reactive approach creates a cycle where regulations perpetually lag emerging scientific evidence, leaving populations exposed to potential risks for decades. The current system may place an undue burden on public agencies to prove harm from mixtures, rather than requiring manufacturers to demonstrate the safety of their products in the context of realistic, mixed exposures.

Addressing this challenge will require more than incremental adjustments to existing frameworks; it necessitates a paradigm shift. The scientific community has been developing advanced tools and approaches, such as exposomics, high-throughput screening, computational toxicology (including QSAR and PBPK modeling), and systems biology, that offer the potential to better characterize exposures, predict interactions, and assess the cumulative risks of chemical mixtures.^67,68,93^ The adoption of new scientific methods into regulatory systems remains slow due to challenges in validation, standardization, and institutional capacity. As innovation advances faster than regulatory adaptation, a gap persists between scientific capability and policy implementation. Integrative frameworks, such as One Health, linking human, animal, and environmental health, provide a useful perspective for addressing complex mixture exposures across ecosystems and species.^17,52,94^ Environmental contaminants often circulate through interconnected ecological systems, accumulating in food webs, altering microbial communities, and impacting wildlife physiology, before reaching humans. For example, pesticide mixtures used in agriculture not only affect soil and aquatic ecosystems but also contribute to antimicrobial resistance and disrupt endocrine function in both livestock and humans. PFAS and other persistent toxicants have been also documented across species and habitats, underlining their global ecological footprint.

Integrating One Health principles into regulatory frameworks could involve cross-sectoral surveillance, ecological risk assessments alongside human toxicology, and early warning systems utilizing sentinel species data. By framing chemical exposures as a multispecies, systems-level problem, One Health enables a more comprehensive and anticipatory approach to chemical safety, bridging regulatory silos and supporting policies that protect entire ecosystems rather than isolated human end points.

The regulatory landscape faces significant challenges in implementation. These dynamics require careful consideration in developing more comprehensive regulatory reforms. Moreover, the principles of environmental justice demand that regulatory reforms prioritize the protection of the most vulnerable and disproportionately exposed communities. This includes ensuring that risk assessments explicitly consider differential exposures and susceptibilities and that affected communities have a meaningful voice in policy development. Addressing these challenges requires a multifaceted approach that balances scientific evidence with practical implementation considerations.

While the task of reforming chemical regulations to adequately address mixtures is daunting, it is not insurmountable. The legislative changes in Canada (CEPA reforms) to incorporate cumulative effects assessments offer promising developments and a potential model for other jurisdictions. The ongoing discussions within the EU regarding the implementation of MAFs also represent a step in the right direction, though their scope and mandatory application remain critical points of development. Some jurisdictions are exploring innovative approaches such as class-based regulations for chemical groups like PFAS, which could provide templates for addressing other chemical classes with similar properties or mechanisms of action. Ultimately, effective reform will require a multipronged strategy that combines scientific innovation, robust policy development, international cooperation, and appropriate application of the precautionary principle balanced with scientific evidence and economic considerations.

It is essential to contextualize current chemical exposures and health outcomes within broader historical trends. While environmental exposure to chemical mixtures is indeed a legitimate concern requiring regulatory attention, by several measures humans are leading healthier and longer lives compared to people born a century ago. This observation does not diminish the importance of addressing mixture toxicity but emphasizes the need for balanced risk communication and evidence-based policy decisions that consider both potential harms and the benefits of chemical technologies in modern society.

## 5 Conclusions

The evidence suggests: the prevailing single-substance approach to chemical regulation may not adequately protect against the complex and interactive risks posed by real-world toxicant mixtures.^7,23^ Despite decades of research showing additive and synergistic effects, most national and international frameworks remain focused on isolated substances, potentially creating a gap in underestimation of cumulative risk, particularly for vulnerable populations. This review shows that challenges between scientific understanding and regulatory action can persist due to fragmented standards, potential industry influence, insufficient precautionary measures, and inadequate data on combined exposures.

To address these challenges, regulatory reform should consider prioritizing mixture risk assessments, harmonized global standards, and robust protections for disproportionately affected communities.^19,31^ Integration of emerging scientific tools, such as exposomics, computational toxicology, and high-throughput screening, along with frameworks like One Health, can enable a shift toward systems-based governance. Regulatory reforms should also incorporate benefit-cost analyses and uncertainty assessments to ensure that protective measures are both scientifically justified and economically feasible. The task is technically and politically complex but important. Without appropriate reform, regulatory systems will continue to lag behind the realities of modern chemical exposure, potentially perpetuating avoidable harm. The need to align policy with science is pressing before cumulative chemical burdens cause further potential damage.^95^

## 6 Recommendations

Given the enormous number of possible chemical combinations and the inherent limitations of regulatory resources, a risk-based prioritization framework is essential. A tiered framework offers a practical means of ranking chemical mixtures based on several key criteria: (a) the frequency and magnitude of coexposure, such as chemicals consistently detected together in large-scale biomonitoring programs like NHANES and HBM4EU, (b) biological plausibility for interactive effects, for instance, when chemicals share molecular targets, metabolic pathways, or similar modes of action, (c) the potential for heightened health risks in vulnerable groups, including children, pregnant individuals, and communities facing disproportionate environmental exposures, and (d) the availability of relevant data and the feasibility of regulatory action. Applying this framework, mixtures of PFAS, organophosphate pesticides, heavy metals, and EDCs should receive the highest priority. This is justified by their frequent co-occurrence in exposed populations, growing evidence of combined toxicological effects, as well as the relative abundance of existing exposure data. Implementing such a stratified approach would enable regulatory bodies to focus research and resources on mixtures with the most significant and addressable public health implications, while ensuring decisions are transparent, reproducible, and evidence-based.

To align chemical governance with the realities of mixture exposure, the following priority actions are proposed:

Mandate science-based mixture risk assessments: consider requiring chemical safety evaluations to routinely include mixture toxicity, especially for high-exposure scenarios (e.g., PFAS, pesticides, EDCs).^31,38^ This should apply to both intentional mixtures (formulations) and common environmental cocontaminants, using a tiered approach based on the exposure data and toxicological concern.

Standardize methodologies and tools: adopt validated, internationally harmonized methods for mixture risk assessment.^67,96^ These should include component-based approaches (e.g., hazard index, toxic equivalency, MAFs), whole-mixture testing where feasible, and integration of high-throughput screening, QSAR, and PBPK models.

Center vulnerable populations: mixture regulation should explicitly protect susceptible groups, children, pregnant women, elderly populations, and socioeconomically marginalized communities, by incorporating additional uncertainty factors, stricter exposure limits, and equity-driven exposure assessments.

Implement science-based precautionary approaches: when interaction data are lacking or inconclusive, consider applying default MAFs based on scientific evidence and encourage manufacturers to demonstrate safety under plausible coexposure scenarios.^34^ Regulatory decisions should be guided by the best available science while acknowledging uncertainties.

Embed One Health into regulatory frameworks: incorporate ecological end points and cross-species vulnerability into mixture assessment strategies, reflecting the interdependence of human, animal, and environmental health. This includes environmental surveillance using sentinel species and cross-sector data integration.^97^

Strengthen transparency and data requirements: consider mandating full public disclosure of mixture-relevant data, including coformulant interactions and environmental persistence; evaluate confidentiality claims for health-impacting data; and develop standardized mixture hazard reporting formats.

Harmonize international standards: promote global consistency through WHO, the Organisation for Economic Co-operation and Development (OECD), UNEP, and other multilateral frameworks; establish global benchmarks for mixture toxicity, including PFAS, metals, and pesticide residues.

Enhance surveillance and biomonitoring: expand national and regional biomonitoring programs to track real-world mixture exposures, with particular attention to hotspots and vulnerable communities. Surveillance data should inform regulatory thresholds and postmarket reassessment triggers.

Fund independent mixture research: increase public investment in mixture toxicology, exposomics, and computational modeling; establish interdisciplinary centers of excellence and train regulators to interpret complex data. Independent funding mechanisms should be prioritized to ensure scientific integrity.

Incorporate comprehensive benefit-cost analysis and uncertainty assessment: regulatory decisions should systematically evaluate the economic costs and benefits of mixture regulations, while explicitly addressing uncertainties in mixture toxicity data. This approach will ensure that protective measures are proportionate to risks and consider the broader societal implications of regulatory actions.

## 7 Limitations

The present review aims at providing a comprehensive assessment of regulatory challenges in addressing toxicant mixtures but several limitations should be noted. First, the literature search was limited to English-language publications from 1980 onward. While this approach captures the most policy-relevant and methodologically standardized research, it can have excluded earlier or non-English studies, particularly those from regions where chemical exposures and regulatory responses differ significantly from high-income countries. Second, although the review includes key examples of toxicant classes (e.g., PFAS, heavy metals, pesticides), it cannot exhaustively cover the full spectrum of relevant mixture scenarios. Notable areas beyond the scope of this review include emerging contaminants such as microplastics, transformation products, and nanomaterials, as well as exposure contexts such as e-waste recycling. These represent important domains for future analysis, especially given their potential to generate complex mixtures. Third, while the review draws on both biomonitoring and toxicological studies, significant uncertainties remain regarding the interactive effects of many mixtures at environmentally relevant doses.^61,97^ Although documented, synergistic effects are rarely observed, and the current scientific understanding of when and how they occur remains incomplete. This highlights the need for continued refinement of mixture assessment methods, including better validation of computational models and expanded access to exposure data sets. Finally, the analysis focuses predominantly on regulatory frameworks and data availability in industrialized regions, notably the US, EU, and Canada. Although global treaties are discussed, a deeper exploration of regulatory capacity and exposure burdens in low- and middle-income countries is lacking. Given the transboundary nature of chemical pollution and the disproportionate exposure risks faced by vulnerable populations in these settings, greater global representation is essential for future work.

Despite these limitations, the review highlights important structural challenges in current chemical governance and provides a clear direction for advancing mixture risk assessment and regulation. Future efforts should focus on bridging data gaps, expanding global inclusion, and adapting regulatory systems to reflect the complexity of real-world exposures.

## Abbreviations

BPA: bisphenol A
CEPA: Canadian Environmental Protection Act
DLC: dioxin-like compounds
EDCs: endocrine-disrupting chemicals
EFSA: European Food Safety Authority
FQPA: Food Quality Protection Act
HBM4EU: European Human Biomonitoring Initiative
HRMS: high-resolution mass spectrometry
MAFs: mixture assessment factors
MCLs: maximum contaminant levels
MRLs: maximum residue limits
NAMs: new approach methodologies
NHANES: National Health and Nutrition Examination Survey
OELs: occupational exposure limits
PBPK: physiologically based pharmacokinetic
PFAS: per- and polyfluoroalkyl substances
PFOA: perfluorooctanoic acid
PFOS: perfluorooctanesulfonic acid
POPs: persistent organic pollutants
QSAR: quantitative structure–activity relationship
REACH: registration
evaluation: authorization and restriction of chemicals
SAICM: Strategic Approach to International Chemicals Management
TEF: toxic equivalency factor
TSCA: Toxic Substances Control Act
WHO: World Health Organization.

## References

[1] Prüss-Ustün A., Wolf J., Corvalán C., Neville T., Bos R., Neira M. (2017). Diseases due to unhealthy environments: an updated estimate of the
global burden of disease attributable to environmental determinants
of health. J. Public Health.

[2] WHO, World Health Organization . An estimated 12.6 million deaths each year are attributable to unhealthy environments, Public Health, Environmental and Social Determinants of Health (PHE), 2016.

[3] Buekers J., David M., Koppen G., Bessems J., Scheringer M., Lebret E., Sarigiannis D., Kolossa-Gehring M., Berglund M., Schoeters G., Trier X. (2018). Development of Policy
Relevant Human Biomonitoring Indicators for Chemical Exposure in the
European Population. Int. J. Environ. Res. Public
Health.

[4] Vermeulen R., Schymanski E. L., Barabási A. L., Miller G. W. (2020). The exposome and
health: Where chemistry meets biology. Science.

[5] Kortenkamp A. (2007). Ten years
of mixing cocktails: a review of combination effects of endocrine-disrupting
chemicals. Environ. Health Perspect..

[6] Kortenkamp A. (2014). Low dose mixture
effects of endocrine disrupters and their implications for regulatory
thresholds in chemical risk assessment. Curr.
Opin. Pharmacol..

[7] Martin O., Scholze M., Ermler S., McPhie J., Bopp S. K., Kienzler A., Parissis N., Kortenkamp A. (2021). Ten years
of research on synergisms and antagonisms in chemical mixtures: A
systematic review and quantitative reappraisal of mixture studies. Environ. Int..

[8] Cedergreen N. (2014). Quantifying
synergy: a systematic review of mixture toxicity studies within environmental
toxicology. PLoS One.

[9] Hamid N., Junaid M., Pei D. S. (2021). Combined
toxicity of endocrine-disrupting
chemicals: A review. Ecotoxicol. Environ. Saf..

[10] Keiter S., Baumann L., Färber H., Holbech H., Skutlarek D., Engwall M., Braunbeck T. (2012). Long-term
effects of a binary mixture
of perfluorooctane sulfonate (PFOS) and bisphenol A (BPA) in zebrafish
(Danio rerio). Aquat. Toxicol..

[11] Zhou R., Cheng W., Feng Y., Wang W., Liang F., Luo F., Yang S., Wang Y. (2020). Combined effects of BPA and PFOS
on fetal cardiac development: In vitro and in vivo experiments. Environ. Toxicol. Pharmacol..

[12] Pyatha S., Kim H., Lee D., Kim K. (2023). Co-exposure to lead, mercury, and
cadmium induces neurobehavioral impairments in mice by interfering
with dopaminergic and serotonergic neurotransmission in the striatum. Front. Public Health.

[13] Wang Y., Chen C., Zhao X., Wang Q., Qian Y. (2015). Assessing
joint toxicity of four organophosphate and carbamate insecticides
in common carp (Cyprinus carpio) using acetylcholinesterase activity
as an endpoint. Pestic. Biochem. Physiol..

[14] Hernández A. F., Gil F., Lacasaña M. (2017). Toxicological
interactions of pesticide
mixtures: an update. Arch. Toxicol..

[15] Claus
Henn B., Coull B. A., Wright R. O. (2014). Chemical mixtures and children’s
health. Curr. Opin. Pediatr..

[16] Zota A. R., Shamasunder B. (2017). The environmental
injustice of beauty: framing chemical
exposures from beauty products as a health disparities concern. Am. J. Obstet. Gynecol..

[17] Ford J. D., King N., Galappaththi E. K., Pearce T., McDowell G., Harper S. L. (2020). The resilience of
indigenous peoples to environmental
change. One Earth.

[18] Makinde O. A., Uthman O. A., Mgbachi I. C., Ichegbo N. K., Sule F. A., Olamijuwon E. O., Okusanya B. O. (2022). Vulnerability in maternal, new-born,
and child health in low- and middle-income countries: Findings from
a scoping review. PLoS One.

[19] Bloch D., Diel P., Epe B., Hellwig M., Lampen A., Mally A., Marko D., Villar Fernández M. A., Guth S., Roth A., Marchan R., Ghallab A., Cadenas C., Nell P., Vartak N., van Thriel C., Luch A., Schmeisser S., Herzler M., Landsiedel R., Leist M., Marx-Stoelting P., Tralau T., Hengstler J. G. (2023). Basic concepts
of mixture toxicity and relevance for risk evaluation and regulation. Arch. Toxicol..

[20] Bopp S. K., Barouki R., Brack W., Dalla Costa S., Dorne J. C. M., Drakvik P. E., Faust M., Karjalainen T. K., Kephalopoulos S., van Klaveren J., Kolossa-Gehring M., Kortenkamp A., Lebret E., Lettieri T., Nørager S., Rüegg J., Tarazona J. V., Trier X., van de Water B., van Gils J., Bergman Å. (2018). Current EU research activities on
combined exposure to multiple chemicals. Environ.
Int..

[21] Land K. L., Ghuneim S. M., Williams B. A., Hannon P. R. (2024). IMPACT OF REAL-LIFE
ENVIRONMENTAL EXPOSURES ON REPRODUCTION: Phthalates disrupt female
reproductive health: a call for enhanced investigation into mixtures. Reproduction.

[22] Bhandare S. D. (2025). Advancements
in toxicological risk assessment: integrating Ferguson’s principle,
computational models, and drug safety guidelines, a comprehensive
framework for improving risk assessment and resource management in
toxicology. Toxicol. Res..

[23] Drakvik E., Altenburger R., Aoki Y., Backhaus T., Bahadori T., Barouki R., Brack W., Cronin M. T. D., Demeneix B., Hougaard Bennekou S., van Klaveren J., Kneuer C., Kolossa-Gehring M., Lebret E., Posthuma L., Reiber L., Rider C., Rüegg J., Testa G., van der Burg B., van der Voet H., Warhurst A. M., van de Water B., Yamazaki K., Öberg M., Bergman Å. (2020). Statement on advancing
the assessment of chemical mixtures and their risks for human health
and the environment. Environ. Int..

[24] Morgan S. E., DeLouise L. A. (2024). Effects of microplastic interaction
with persistent
organic pollutants on the activity of the aryl hydrocarbon and estrogen
receptors. Chemosphere.

[25] Vorkamp K., Castaño A., Antignac J. P., Boada L. D., Cequier E., Covaci A., Esteban López M., Haug L. S., Kasper-Sonnenberg M., Koch H. M., Pérez Luzardo O., Osi̅te A., Rambaud L., Pinorini M. T., Sabbioni G., Thomsen C. (2021). Biomarkers, matrices and analytical methods targeting
human exposure to chemicals selected for a European human biomonitoring
initiative. Environ. Int..

[26] Wild C. P. (2012). The exposome:
from concept to utility. Int. J. Epidemiol..

[27] Braun J. M., Gennings C., Hauser R., Webster T. F. (2016). What Can Epidemiological
Studies Tell Us about the Impact of Chemical Mixtures on Human Health?. Environ. Health Perspect..

[28] Weisskopf M. G., Seals R. M., Webster T. F. (2018). Bias Amplification in Epidemiologic
Analysis of Exposure to Mixtures. Environ. Health
Perspect..

[29] Woodruff T. J., Zota A. R., Schwartz J. M. (2011). Environmental
chemicals in pregnant
women in the United States: NHANES 2003–2004. Environ. Health Perspect..

[30] Cattaneo I., Kalian A. D., Di Nicola M. R., Dujardin B., Levorato S., Mohimont L., Nathanail A. V., Carnessechi E., Astuto M. C., Tarazona J. V., Kass G. E. N., Liem A. K. D., Robinson T., Manini P., Hogstrand C., Price P. S., Dorne J. L. C. M. (2023). Risk Assessment of Combined Exposure
to Multiple Chemicals at the European Food Safety Authority: Principles,
Guidance Documents, Applications and Future Challenges. Toxins (Basel).

[31] More S. J., Bampidis V., Benford D., Bragard C., Hernandez-Jerez A., Bennekou S. H., Halldorsson T. I., Koutsoumanis K. P., Lambré C., Machera K., Naegeli H., Nielsen S. S., Schlatter J. R., Schrenk D., Silano V., Turck D., Younes M., Benfenati E., Crépet A., Te Biesebeek J. D., Testai E., Dujardin B., Dorne J. L. C., Hogstrand C., EFSA Scientific Committee (2021). Guidance Document on Scientific criteria
for grouping chemicals into assessment groups for human risk assessment
of combined exposure to multiple chemicals. EFSA J..

[32] Hill C. E., Myers J. P., Vandenberg L. N. (2018). Nonmonotonic
Dose-Response Curves
Occur in Dose Ranges That Are Relevant to Regulatory Decision-Making. Dose Response.

[33] Koman P. D., Singla V., Lam J., Woodruff T. J. (2019). Population
susceptibility:
A vital consideration in chemical risk evaluation under the Lautenberg
Toxic Substances Control Act. PLoS Biol..

[34] Krimsky S. (2017). The unsteady
state and inertia of chemical regulation under the US Toxic Substances
Control Act. PLoS Biol..

[35] Rayasam S. D. G., Koman P. D., Axelrad D. A., Woodruff T. J., Chartres N. (2022). Toxic Substances
Control Act (TSCA) Implementation: How the Amended Law Has Failed
to Protect Vulnerable Populations from Toxic Chemicals in the United
States. Environ. Sci. Technol..

[36] McPartland J., Shaffer R. M., Fox M. A., Nachman K. E., Burke T. A., Denison R. A. (2022). Charting a Path
Forward: Assessing the Science of Chemical
Risk Evaluations under the Toxic Substances Control Act in the Context
of Recent National Academies Recommendations. Environ. Health Perspect..

[37] US EPA (US Environmental Protection Agency) Assessing and Managing Chemicals under TSCA, 2023. Available from: https://www.epa.gov/assessing-and-managing-chemicals-under-tsca (accessed June 30, 2025).

[38] Treu G., Schulze J., Galert W., Hassold E. (2024). Regulatory and practical
considerations on the implementation of a mixture allocation factor
in REACH. Environ. Sci. Europe.

[39] ECHA (European Chemicals Agency) . Understanding REACH, 2023. Available from: https://echa.europa.eu/regulations/reach/understanding-reach (accessed June 30, 2025).

[40] Toda E., Have C. T., Pacyna J. M. (2020). The Minamata
Convention: A tool for
global regulation of mercury pollution. Chem.
Int..

[41] Wang Z., Adu-Kumi S., Diamond M. L., Guardans R., Harner T., Harte A., Kajiwara N., Klánová J., Liu J., Moreira E. G., Muir D. C. G., Suzuki N., Pinas V., Seppälä T., Weber R., Yuan B. (2022). Enhancing
Scientific Support for the Stockholm Convention’s Implementation:
An Analysis of Policy Needs for Scientific Evidence. Environ. Sci. Technol..

[42] US EPA (US Environmental Protection Agency) Organophosphate Pesticides: Revised OP Cumulative Risk Assessment; Office of Pesticide Programs: Washington, DC, 2002.

[43] US EPA (US Environmental Protection Agency) Revised N-methyl Carbamate Cumulative Risk Assessment; Office of Pesticide Programs: Washington, DC, 2007.

[44] Carrasco
Cabrera L., Di Piazza G., Dujardin B., Medina Pastor P., European Food Safety Authority EFSA (2023). The 2021 European Union report on pesticide residues
in food. EFSA J..

[45] Richardson S. D., Kimura S. Y. (2019). Water Analysis: Emerging Contaminants
and Current Issues. Anal. Chem..

[46] Escher B. I., Hackermüller J., Polte T., Scholz S., Aigner A., Altenburger R., Böhme A., Bopp S. K., Brack W., Busch W., Chadeau-Hyam M., Covaci A., Eisenträger A., Galligan J. J., Garcia-Reyero N., Hartung T., Hein M., Herberth G., Jahnke A., Kleinjans J., Klüver N., Krauss M., Lamoree M., Lehmann I., Luckenbach T., Miller G. W., Müller A., Phillips D. H., Reemtsma T., Rolle-Kampczyk U., Schüürmann G., Schwikowski B., Tan Y. M., Trump S., Walter-Rohde S., Wambaugh J. F. (2017). From the exposome to mechanistic understanding of chemical-induced
adverse effects. Environ. Int..

[47] Hernández A. F., Tsatsakis A. M. (2017). Human exposure to chemical mixtures:
Challenges for
the integration of toxicology with epidemiology data in risk assessment. Food Chem. Toxicol..

[48] Landrigan P. J., Goldman L. R. (2011). Children’s
vulnerability to toxic chemicals:
a challenge and opportunity to strengthen health and environmental
policy. Health Aff. (Millwood).

[49] Maffini M. V., Vandenberg L. N. (2025). Editorial:
Emerging topics on chemical safety assessment. Front. Toxicol..

[50] Chentouf A. (2022). Toxicological
Evaluation of Complex Mixtures: Prediction and interactions--A review. J. Pharm. Pharmacol. Res..

[51] UNEP (United Nations Environment Programme) . Basel Convention: Technical Guidelines, 2023. Available from: https://www.basel.int/Implementation/TechnicalGuidelines (accessed June 30, 2025).

[52] UNEP (United Nations Environment Programme) . Global Framework on Chemicals -- Towards a Planet Free of Harm from Chemicals and Waste, 2024. Available from: https://www.unep.org/(accessed June 29, 2025).

[53] Parliament of Canada . Bill S-5: An Act to amend the Canadian Environmental Protection Act, 1999, to make related amendments to the Food and Drugs Act and to repeal the Perfluorooctane Sulfonate Virtual Elimination Act, 2023. Available from: https://www.parl.ca/legisinfo/en/bill/44-1/s-5 (accessed June 27, 2025).

[54] Richardson S. D., Ternes T. A. (2022). Water Analysis:
Emerging Contaminants and Current Issues. Anal.
Chem..

[55] Kaufman J. A., Wright J. M., Evans A., Rivera-Núñez Z., Meyer A., Reckhow D. A., Narotsky M. G. (2024). Risks of obstructive
genitourinary birth defects in relation to trihalomethane and haloacetic
acid exposures: expanding disinfection byproduct mixtures analyses
using relative potency factors. J. Expo. Sci.
Environ. Epidemiol..

[56] EEA (European Environment Agency) . Air quality in Europe 2023 report, 2023. Available from: https://www.eea.europa.eu/publications/(accessed June 28, 2025).

[57] Šolc M., Blaško P., Girmanová L., Kliment J. (2022). The Development Trend
of the Occupational Health and Safety in the Context of ISO 45001:2018. Standards.

[58] European Commission (EC) . Chemicals Strategy for Sustainability Towards a Toxic-Free Environment, 2023. Available from https://environment.ec.europa.eu/strategy/chemicals-strategy_en (accessed: July 2, 2025).

[59] Lagunas-Rangel F. A., Linnea-Niemi J. V., Kudłak B., Williams M. J., Jönsson J., Schiöth H. B. (2022). Role of the Synergistic Interactions of Environmental
Pollutants in the Development of Cancer. Geohealth.

[60] Lambert J. C., Lipscomb J. C. (2007). Mode of action as a determining factor
in additivity
models for chemical mixture risk assessment. Regul. Toxicol. Pharmacol..

[61] Martin O. V. (2023). Synergistic
effects of chemical mixtures: How frequent is rare?. Curr. Opin. Toxicol..

[62] Kortenkamp A., Altenburger R. (1999). Synergisms with mixtures of xenoestrogens: a reevaluation
using the method of isoboles. Sci. Total Environ..

[63] Vandenberg L. N., Colborn T., Hayes T. B., Heindel J. J., Jacobs D. R., Lee D. H., Shioda T., Soto A. M., vom Saal F. S., Welshons W. V., Zoeller R. T., Myers J. P. (2012). Hormones
and endocrine-disrupting chemicals: low-dose effects and nonmonotonic
dose responses. Endocr. Rev..

[64] Hertzberg R. C., MacDonell M. M. (2002). Synergy and other ineffective mixture
risk definitions. Sci. Total Environ..

[65] Kortenkamp A. (2022). Invited Perspective:
How Relevant Are Mode-of-Action Considerations for the Assessment
and Prediction of Mixture Effects?. Environ.
Health Perspect..

[66] Schmeisser S., Miccoli A., von Bergen M., Berggren E., Braeuning A., Busch W., Desaintes C., Gourmelon A., Grafström R., Harrill J., Hartung T., Herzler M., Kass G. E. N., Kleinstreuer N., Leist M., Luijten M., Marx-Stoelting P., Poetz O., van Ravenzwaay B., Roggeband R., Rogiers V., Roth A., Sanders P., Thomas R. S., Marie Vinggaard A., Vinken M., van de Water B., Luch A., Tralau T. (2023). New approach methodologies in human
regulatory toxicology - Not if, but how and when. Environ. Int..

[67] Belfield S. J., Firman J. W., Enoch S. J., Madden J. C., Erik
Tollefsen K., Cronin M. T. (2023). A review of quantitative structure-activity
relationship modelling approaches to predict the toxicity of mixtures. Comput. Toxicol..

[68] Muratov E. N., Bajorath J., Sheridan R. P., Tetko I. V., Filimonov D., Poroikov V., Oprea T. I., Baskin I. I., Varnek A., Roitberg A., Isayev O., Curtalolo S., Fourches D., Cohen Y., Aspuru-Guzik A., Winkler D. A., Agrafiotis D., Cherkasov A., Tropsha A. (2020). QSAR without borders. Chem. Soc.
Rev..

[69] Rappaport S. M. (2011). Implications
of the exposome for exposure science. J. Expo.
Sci. Environ. Epidemiol..

[70] Hartung, T. ; Navas-Acien, A. ; Chiu, W. A. Future Directions Workshop: Advancing the Next Scientific Revolution in Toxicology Workshop Report, 2023. Available from: https://basicresearch.defense.gov/Programs/Future-Directions-Workshops (accessed June 1, 2025).

[71] Sexton K., Hattis D. (2007). Assessing cumulative
health risks from exposure to
environmental mixtures - three fundamental questions. Environ. Health Perspect..

[72] Handford C. E., Elliott C. T., Campbell K. (2015). A review of the global pesticide
legislation and the scale of challenge in reaching the global harmonization
of food safety standards. Integr. Environ. Assess.
Manage..

[73] Kwiatkowski C. F., Andrews D. Q., Birnbaum L. S., Bruton T. A., DeWitt J. C., Knappe D. R. U., Maffini M. V., Miller M. F., Pelch K. E., Reade A., Soehl A., Trier X., Venier M., Wagner C. C., Wang Z., Blum A. (2020). Scientific Basis for
Managing PFAS as a Chemical Class. Environ.
Sci. Technol. Lett..

[74] Robinson C., Portier C. J., Čavoški A., Mesnage R., Roger A., Clausing P., Whaley P., Muilermann H., Lyssimachou A. (2020). Achieving a High Level of Protection
from Pesticides
in Europe: Problems with the Current Risk Assessment Procedure and
Solutions. Eur. J. Risk Regul..

[75] US EPA (US Environmental Protection Agency) Cumulative Assessment of Risk from Pesticides, 2023b. Available from: https://www.epa.gov/pesticide-science-and-assessing-pesticide-risks/cumulative-assessment-risk-pesticides (accessed June15, 2025).

[76] Panieri E., Baralic K., Djukic-Cosic D., Buha Djordjevic A., Saso L. (2022). PFAS Molecules: A Major Concern for
the Human Health and the Environment. Toxics.

[77] Domingo J. L., Nadal M. (2025). PCDD/Fs in human tissues: A review
of global biomonitoring data. Chemosphere.

[78] Nadal M., Domingo J. L. (2025). Non-Invasive Matrices
for the Human Biomonitoring of
PFAS: An Updated Review of the Scientific Literature. Toxics.

[79] Ješeta M., Navrátilová J., Franzová K., Fialková S., Kempisty B., Ventruba P., Žáková J., Crha I. (2021). Overview of the Mechanisms
of Action of Selected Bisphenols and Perfluoroalkyl
Chemicals on the Male Reproductive Axes. Front.
Genet..

[80] Barry V., Winquist A., Steenland K. (2013). Perfluorooctanoic
acid (PFOA) exposures
and incident cancers among adults living near a chemical plant. Environ. Health Perspect..

[81] Chang Z., Qiu J., Wang K., Liu X., Fan L., Liu X., Zhao Y., Zhang Y. (2023). The relationship
between co-exposure
to multiple heavy metals and liver damage. J.
Trace Elem. Med. Biol..

[82] Dórea J. G. (2020). Neurotoxic
effects of combined exposures to aluminum and mercury in early life
(infancy). Environ. Res..

[83] Ouyang L., Li Q., Rao S., Su R., Zhu Y., Du G., Xie J., Zhou F., Feng C., Fan G. (2023). Cognitive outcomes
caused by low-level lead, cadmium, and mercury mixture exposure at
distinct phases of brain development. Food Chem.
Toxicol..

[84] Yin G., Zhao S., Zhao M., Xu J., Ge X., Wu J., Zhou Y., Liu X., Wei L., Xu Q. (2024). Joint and
interactive effects of metal mixtures on liver damage: Epidemiological
evidence from repeated-measures study. Ecotoxicol.
Environ. Saf..

[85] Jacobson P. D., Boufides C. H., Chrysler D., Bernstein J., Citrin T. (2020). The Role of the Legal System in the
Flint Water Crisis. Milbank Q.

[86] Ruckart P. Z., Ettinger A. S., Hanna-Attisha M., Jones N., Davis S. I., Breysse P. N. (2019). The Flint Water
Crisis: A Coordinated Public Health
Emergency Response and Recovery Initiative. J. Public Health Manag. Pract..

[87] Hanna-Attisha M., LaChance J., Sadler R. C., Champney
Schnepp A. (2016). Elevated Blood
Lead Levels in Children Associated With the Flint Drinking Water Crisis:
A Spatial Analysis of Risk and Public Health Response. Am. J. Public Health.

[88] Ezell J. M., Bhardwaj S., Chase E. C. (2023). Child Lead Screening
Behaviors and
Health Outcomes Following the Flint Water Crisis. J. Racial Ethn. Health Disparities.

[89] Michaels R. A. (2020). Legacy
contaminants of emerging concern: Lead (Pb), flint (MI), and human
health. Environ. Claims J..

[90] Ahmad M. F., Ahmad F. A., Alsayegh A. A., Zeyaullah M., AlShahrani A. M., Muzammil K., Saati A. A., Wahab S., Elbendary E. Y., Kambal N., Abdelrahman M. H., Hussain S. (2024). Pesticides impacts on human health and the environment
with their mechanisms of action and possible countermeasures. Heliyon.

[91] Hernández A. F., Parrón T., Tsatsakis A. M., Requena M., Alarcón R., López-Guarnido O. (2013). Toxic effects
of pesticide mixtures
at a molecular level: their relevance to human health. Toxicology.

[92] Zhang Y., Kiljanek T., Rundlöf M., Brunelli M., Mazzacano C. A., van der Sluijs J. P., Nocelli R. C. (2022). Pesticide mixture toxicity
in field-collected honey bees: Analysis of the influence of chemical
factors on adverse health outcomes. Sci. Total
Environ..

[93] Judson R., Richard A., Dix D. J., Houck K., Martin M., Kavlock R., Dellarco V., Henry T., Holderman T., Sayre P., Tan S., Carpenter T., Smith E. (2009). The toxicity data landscape for environmental
chemicals. Environ. Health Perspect..

[94] Wang F., Xiang L., Sze-Yin
Leung K., Elsner M., Zhang Y., Guo Y., Pan B., Sun H., An T., Ying G., Brooks B. W., Hou D., Helbling D. E., Sun J., Qiu H., Vogel T. M., Zhang W., Gao Y., Simpson M. J., Luo Y., Chang S. X., Su G., Wong B. M., Fu T. M., Zhu D., Jobst K. J., Ge C., Coulon F., Harindintwali J. D., Zeng X., Wang H., Fu Y., Wei Z., Lohmann R., Chen C., Song Y., Sanchez-Cid C., Wang Y., El-Naggar A., Yao Y., Huang Y., Cheuk-Fung Law J., Gu C., Shen H., Gao Y., Qin C., Li H., Zhang T., Corcoll N., Liu M., Alessi D. S., Li H., Brandt K. K., Pico Y., Gu C., Guo J., Su J., Corvini P., Ye M., Rocha-Santos T., He H., Yang Y., Tong M., Zhang W., Suanon F., Brahushi F., Wang Z., Hashsham S. A., Virta M., Yuan Q., Jiang G., Tremblay L. A., Bu Q., Wu J., Peijnenburg W., Topp E., Cao X., Jiang X., Zheng M., Zhang T., Luo Y., Zhu L., Li X., Barceló D., Chen J., Xing B., Amelung W., Cai Z., Naidu R., Shen Q., Pawliszyn J., Zhu Y. G., Schaeffer A., Rillig M. C., Wu F., Yu G., Tiedje J. M. (2024). Emerging contaminants: A One Health perspective. Innovation (Camb.).

[95] Rogiers V., Herrmann K., Holley T., Holzhutter H. G., Jaworska J., Kleinjans J., Knudsen L., Kolle S., Marx U., Niemela J., Prieto P., Rovida C., Benfenati E. (2023). The way forward for assessing the human health safety
of cosmetics in the EU - Workshop proceedings. Toxicology.

[96] OECD, Organization for Economic Co-operation and Development , Considerations for Assessing the Risks of Combined Exposure to Multiple Chemicals, OECD Series on Testing and Assessment, 2018. Available at: https://www.oecd.org/en/publications/considerations-for-assessing-the-risks-of-combined-exposure-to-multiple-chemicals_ceca15a9-en.html (accessed June 28, 2025).

[97] Tegegne H.
A., Freeth F. T., Bogaardt C., Taylor E., Reinhardt J., Collineau L., Prada J. M., Hénaux V. (2024). Implementation
of One Health surveillance systems: Opportunities and challenges -
lessons learned from the OH-EpiCap application. One Health.

